# Supplementary material for: Combinatorial allosteric modulation of agonist response in a self-interacting G-protein coupled receptor
Source: Commun Biol. 2020 Jan 15;3:27. doi: 10.1038/s42003-020-0752-4 (PMC6962373; doi:10.1038/s42003-020-0752-4)

Supplementary Figure 1

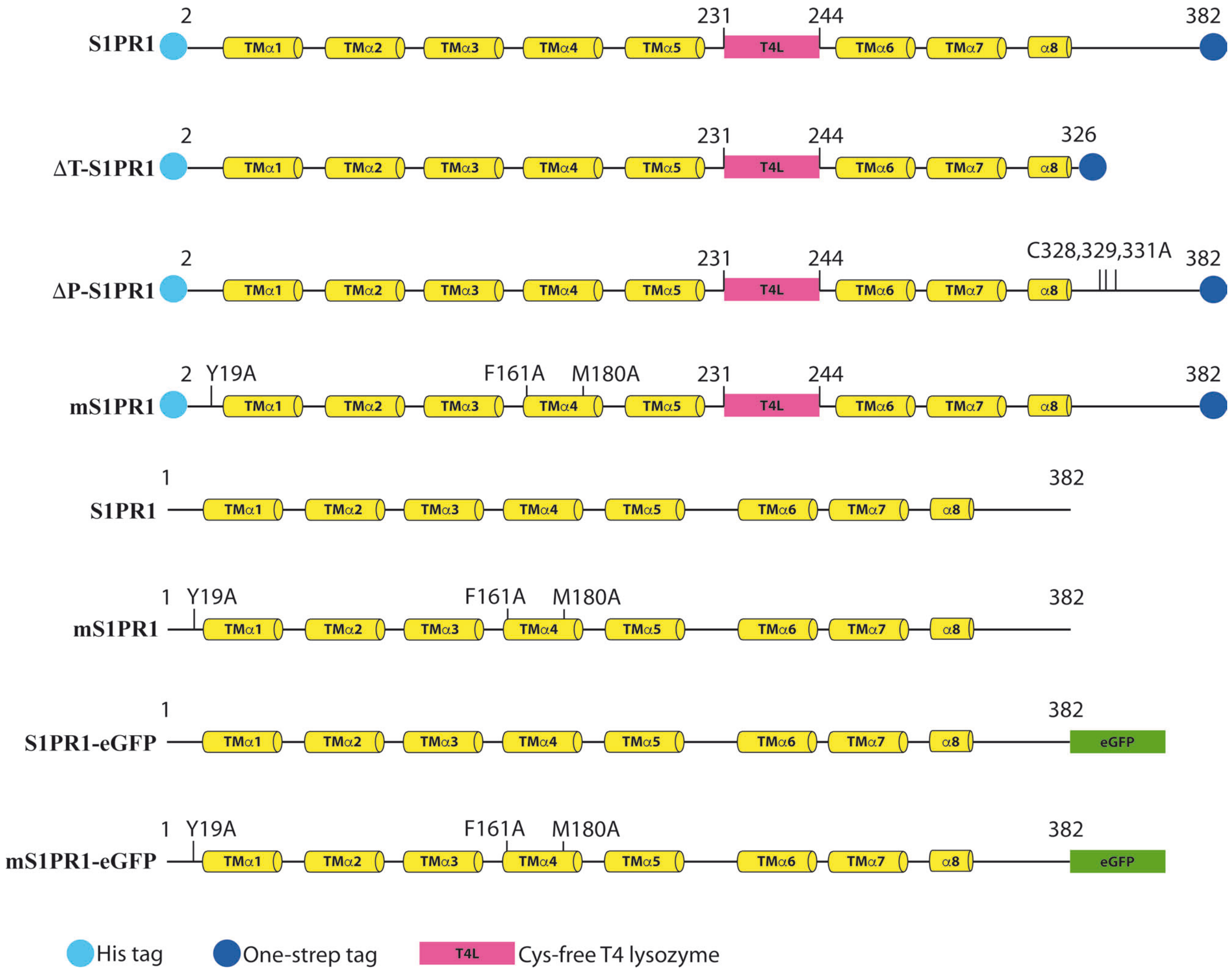

Supplementary Figure 1 | S1PR1 constructs. Schematic representation of the S1PR1 variants used in this work.

Supplementary Figure 2

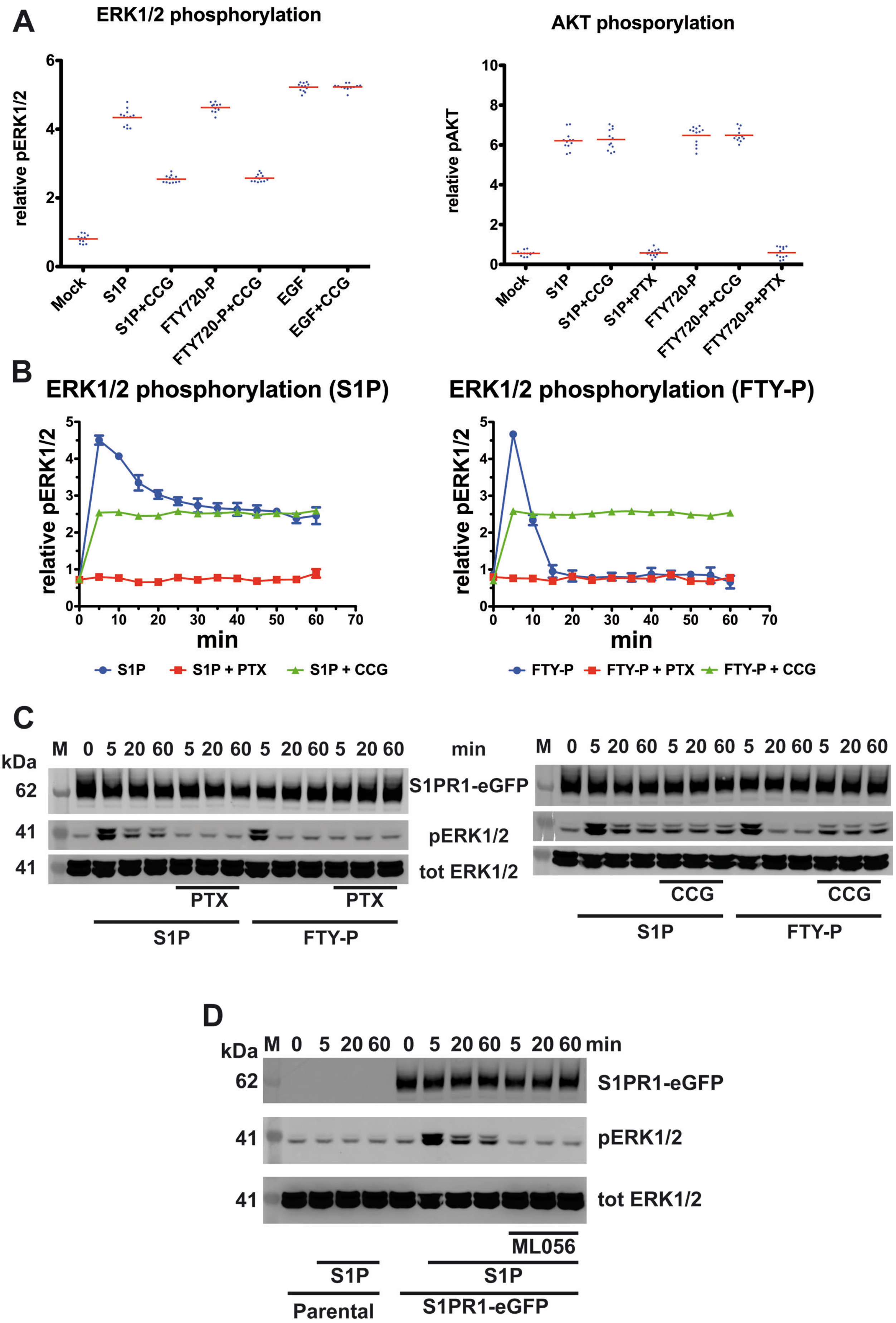

Supplementary Figure 2 | Effects of S1PR1 overexpression in HEK293 cells. (a) HEK293 S1PR1 expressing cells were S1P-starved for 24 hours, with or without 300 ng/mL pertussis toxin (PTX) for 18 hours and with 0.1% DMSO alone or with 50 µM CCG215022 (CCG) for 30 min prior of being stimulated with either S1P or FTY720-P at 0.1 µM or with 20 nM EGF for 5 min. pERK1/2 or pAKT were measured in the cell extracts by ELISA and expressed as relative units; n=12 independent experiments. **(b)** HEK293 cells expressing S1PR1-eGFP were S1P-starved for 24 hours and treated as in (a) and stimulated with 0.1 µM S1P or FTY720-P for the indicated times. pERK1/2 was measured in the cell extracts by ELISA and expressed as relative units; n=3 independent experiments. **(c)** Protein extracts from cells treated as in (a) for the indicated times were assayed by immunoblot for S1PR1, ERK1/2 and pERK1/2. **(d)** Parental HEK293 or S1PR1-eGFP expressing cells were S1P-starved for 24 hours and then stimulated for the indicated times with 0.1 µM S1P with or without 30 min pre-treatment with 100 µM ML056. Cell extracts were then analysed in immunoblotting for S1PR1, ERK1/2 and pERK1/2.

Supplementary Figure 3

A

PISA				FOLDX																			
RESIDUE	BSA ($Å^2$)	SE (kcal/mol)	% of total SE	ΔΔG [mut-wt] (kcal/mol)																			
				A	C	D	E	F	G	H	I	K	L	M	N	P	Q	R	S	T	V	W	Y
Val16	39.1	0.63	7.6	-0.03	-0.06	-0.21	-0.30	-0.32	-0.11	-0.24	0.05	-0.01	-0.10	-0.37	-0.08	-0.07	-0.05	-0.01	-0.06	0.01	0.00	-0.22	-0.26
Ser17	22.7	0.17	2.1	0.39	0.40	0.58	-0.11	-0.48	-0.26	0.77	-0.08	-0.21	-0.16	0.31	0.60	-0.31	-0.13	-0.21	0.00	0.58	0.52	0.48	-0.54
Asp18	81.9	-0.55	-6.7	0.57	0.25	0.00	0.79	0.75	1.00	1.04	0.50	-0.08	-0.89	-0.41	0.32	0.49	1.01	-0.39	1.02	0.78	0.49	0.47	0.67
Tyr19	86.4	0.52	6.3	1.39	0.24	1.46	1.18	-0.58	1.87	1.02	0.12	1.53	0.39	0.13	1.62	1.00	1.04	1.53	1.25	1.24	0.30	0.47	0.00
Val20	31.5	0.50	6.1	0.26	0.24	0.90	0.52	0.39	0.36	-0.33	-0.09	-0.29	0.04	-0.16	0.34	0.52	-0.04	-0.29	0.50	0.19	0.00	0.04	0.18
Trp71	23.2	0.24	2.9	0.38	0.67	0.38	0.12	-0.05	0.46	0.43	0.44	0.70	0.42	-0.03	0.46	0.49	0.31	0.81	0.42	0.72	0.60	0.00	0.17
Tyr82	6.6	0.11	1.3	0.00	0.03	-0.87	0.06	-0.38	0.00	-0.17	-0.02	0.03	-0.12	-0.25	-0.02	0.01	0.01	-0.04	-1.05	0.00	0.01	-0.29	0.00
Ala115	36.3	0.53	6.5	0.00	0.08	1.29	1.00	-0.23	0.33	0.62	-0.15	0.45	-0.01	-0.22	0.71	-0.06	0.24	-0.04	0.32	-0.05	-0.42	-0.11	-0.05
Leu119	30.8	0.49	6.0	1.19	0.91	0.15	1.00	0.56	0.65	0.02	0.34	0.66	0.00	-0.02	1.18	1.32	0.95	0.77	1.35	1.37	0.81	0.07	0.36
Phe158	62.8	1.00	12.2	0.94	0.77	0.72	0.65	-0.04	1.06	0.73	-0.23	1.09	-0.31	-0.12	1.01	2.12	0.76	1.20	1.18	1.08	0.58	-1.08	0.00
Phe161	75.7	1.21	14.7	1.97	1.68	1.71	2.29	0.00	2.01	1.56	0.04	2.17	-0.21	0.60	1.30	1.18	2.14	2.27	2.11	3.17	0.94	-0.84	1.00
Ser165	14.0	-0.16	-1.9	0.00	-0.18	-0.02	0.11	-1.44	0.02	-0.32	-0.45	-0.04	-0.69	-1.46	0.02	-0.13	0.03	-0.03	0.00	-0.02	-0.25	-1.51	-0.52
Val169	5.9	0.09	1.1	0.07	0.02	0.11	-0.14	-0.96	0.05	-0.16	-0.14	-0.05	-0.39	-0.41	0.01	0.07	-0.01	-0.28	0.07	0.07	0.00	-1.69	-0.60
Leu172	32.3	0.52	6.3	0.39	0.42	0.31	0.31	0.11	0.48	0.32	0.13	0.21	0.00	-0.16	0.35	0.39	0.27	0.31	0.44	0.35	0.31	-0.52	0.19
Ile179	49.3	0.73	8.9	0.40	0.54	1.75	1.51	7.03	0.71	0.60	0.04	1.20	0.08	0.10	1.69	0.66	0.93	0.64	0.84	0.37	0.11	0.38	0.71
Met180	89.4	1.72	20.9	1.13	0.68	1.65	1.38	0.90	2.02	2.93	0.85	0.81	0.39	0.00	1.54	1.10	1.28	1.31	2.08	0.70	0.55	0.43	1.60
Gly181	32.3	0.23	2.8	-0.32	-0.63	-0.30	-1.14	-0.70	0.00	-0.46	-1.16	-1.24	-1.56	-1.96	-0.16	0.67	-0.98	-0.68	0.05	1.17	-0.56	-0.40	-0.62
Asn183	18.0	-0.27	-3.2	0.36	0.22	1.06	0.85	-0.45	0.31	0.13	0.06	0.37	-0.17	0.09	0.00	0.38	-0.03	0.42	0.14	0.07	0.13	-1.29	-0.09
Cys184	2.2	0.03	0.4	-0.31	0.00	0.08	0.07	-0.35	-0.04	-0.08	-0.34	-0.47	-0.31	-0.32	-0.28	-0.36	-0.30	-0.88	-0.33	-0.23	-0.29	-0.32	-0.32
Ile185	6.2	0.08	1.0	0.23	0.48	0.57	0.51	0.18	0.46	0.03	0.00	0.29	0.04	0.14	0.40	0.49	0.34	-0.10	-0.86	-0.99	-0.04	-0.04	0.21
Ser186	40.8	0.31	3.7	0.01	0.03	0.78	0.60	-1.09	0.21	0.56	-0.46	-0.34	-0.45	-0.12	-0.10	2.87	-0.16	-0.42	0.00	0.68	0.18	-0.37	-0.73
Ala187	6.0	0.09	1.1	0.00	-0.07	0.33	0.25	-0.16	0.15	-1.09	-0.23	-0.96	-0.17	-0.17	-0.12	0.44	-0.67	-0.56	0.09	0.10	-0.14	-0.22	-0.15

B

ΔG_{dim} (kcal/mol)	
WT S1PR1	-14.4
mS1PR1	-7.3

Supplementary Figure 3 | Structural analysis of the S1PR1 dimer. (a) The dimer structure was analysed using the PISA server to computed buried surface area (BSA), solvation energy (SE), and the relative contribution of each amino acid at the interface (residues contributing more that 5% of the total computed solvation energy are highlighted in green). Energy calculations were performed with FoldX. Each amino acid at the dimer interface was mutated to the other 19 amino acids (shown in single letter code), and the variation in dimerization energy ($\Delta\Delta G_{dim}$) was computed assuming a membrane-embedded protein. Positive values indicate that the mutation is expected to destabilize the quaternary assembly. Highlighted in red are the $\Delta\Delta G_{dim}$ values associated with the mutations engineered to obtain the mS1PR1 construct. **(b)** Comparison of the ΔG_{dim} for the wild type S1PR1 and the Tyr19Ala/Phe161Ala/Met180Ala (mS1PR1) triple mutant, computed using FoldX, showing the expected destabilizing effect of the mutations.

Supplementary Figure 4

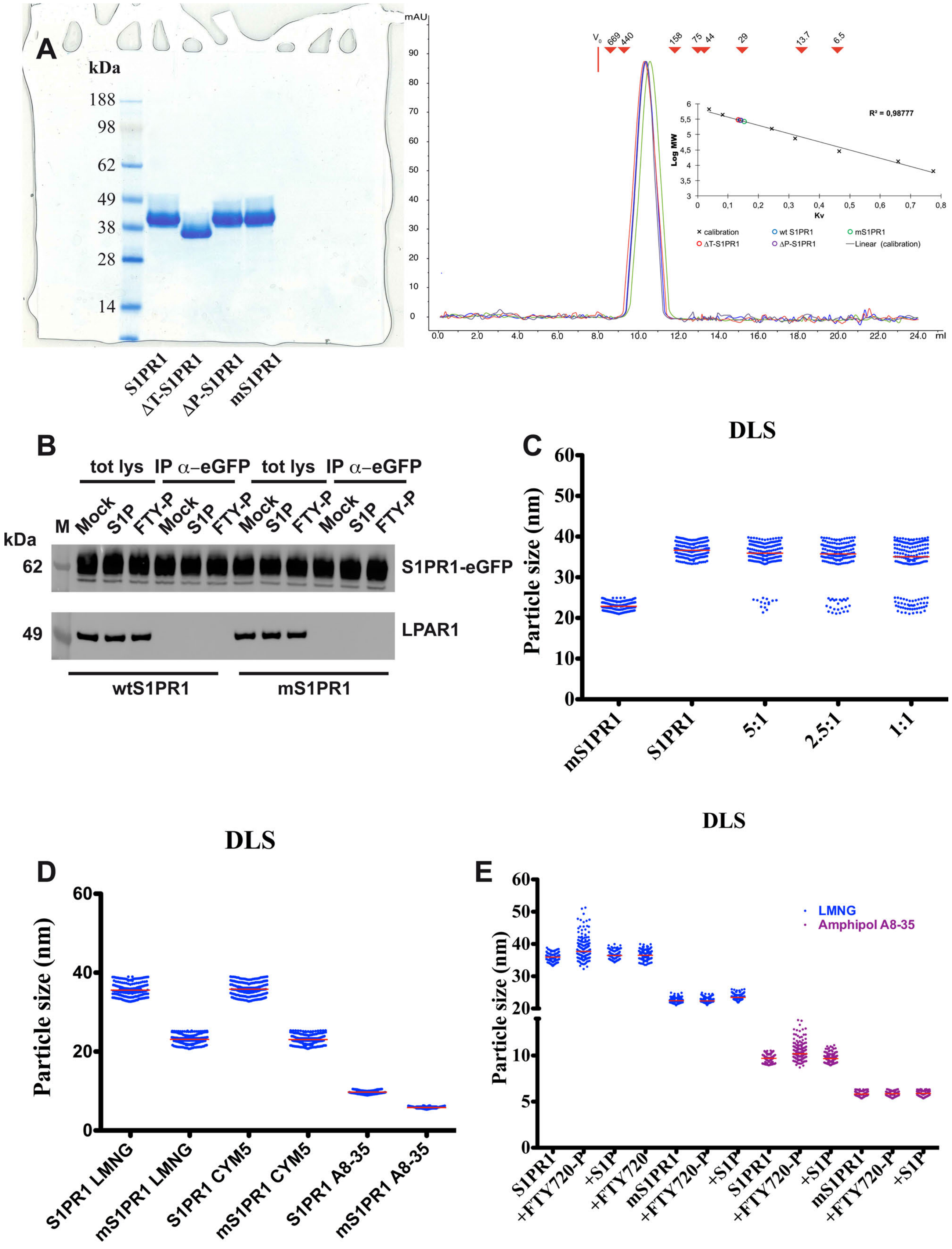

Supplementary Figure 4 | Analysis of S1PR1 self-interaction. (a) Coomassie-stained SDS-PAGE and SEC analysis of the recombinant purified S1PR1 variants produced in insect cells used in this work. **(b)** HEK293 cells expressing S1PR1-eGFP were S1P-starved for 24 hours and stimulated with 0.1 μM S1P or FTY720-P for 5; anti-GFP immunoprecipitation was performed on the cell extracts and the resin eluates analysed by immunoblotting for S1PR1 and LPAR1. **(c)** Hydrodynamic radii of purified S1PR1, mS1PR1 or mS1PR1:S1PR1 mixtures at the indicated molar ratios were measured by DLS at 12.5 μM final protein concentration; individual proteins or the protein mixtures were incubated for 30 min at 37°C prior of being read at the same temperature for 10 min with 10 sec reads; the experiments were performed in triplicates and pooled; single reads for each condition are shown in the plot; n= 175 reads; red horizontal bars indicate the median values. **(d)** DLS measures of S1PR1 or mS1PR1 incubated, read and plotted as in (c) in the indicated detergent systems (LMNG: lauryl maltose neopentyl glycol, CYM5: cymal 5, A8-35: Amphipol 8-35). n= 175 reads. 95% CI intervals: S1PR1 LMNG=35.3-35.9 nm, mS1PR1 LMNG=22.9-23.3 nm, S1PR1 CYM5=35.5-36 nm, mS1PR1 CYM5=22.9-23.3 nm, S1PR1 A8-35=9.7-9.8 nm, mS1PR1 A8-35=5.8-5.9 nm **(e)** DLS measures of S1PR1 or mS1PR1 incubated, read and plotted as in (c) with the indicated detergent systems and the test ligands at 150 μM. n= 175 reads. 95% CI intervals: S1PR1 LMNG=35.7-36.1 nm, S1PR1 LMNG + FTY720-P=38-39 nm, S1PR1 LMNG + S1P=36.4-36.7 nm, S1PR1 LMNG + FTY720=36.4-36.8 nm, mS1PR1 LMNG=22.4-22.6 nm, mS1PR1 LMNG + FTY720-P=22.4-22.7 nm, mS1PR1 LMNG + S1P=23.4-23.7 nm, S1PR1 A8-35=9.6-9.8 nm, S1PR1 A8-35 + FTY720-P=10.3-10.5 nm, S1PR1 A8-35 + S1P=9.6-9.8 nm, mS1PR1 A8-35=5.8-5.9 nm, mS1PR1 A8-35 + FTY720-P=5.8-5.9 nm, mS1PR1 A8-35 + S1P=5.8-5.9 nm.

Supplementary Figure 5

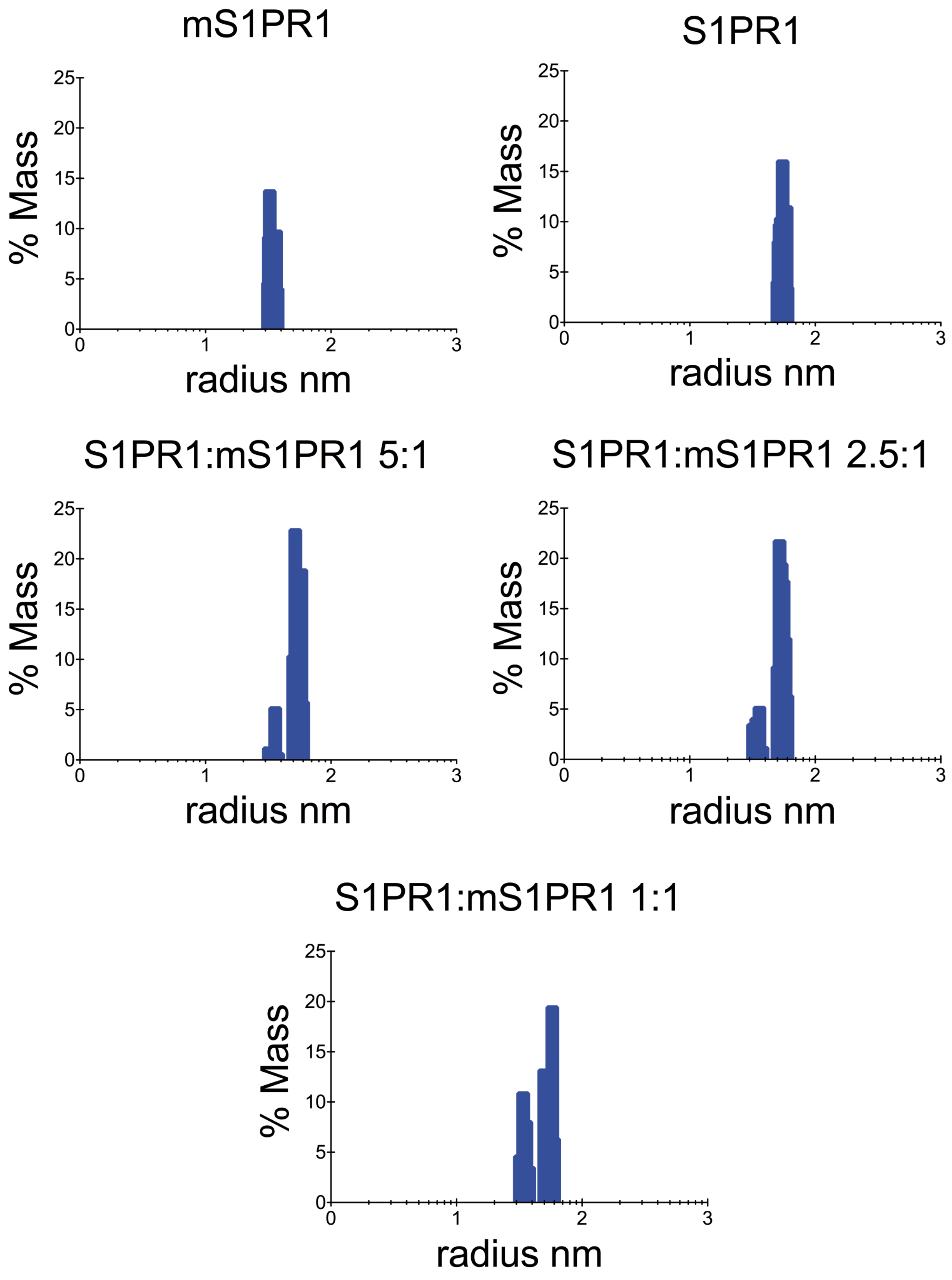

Supplementary Figure 5 | Frequency distributions of DLS experiments showed in Fig. S4 C.

Supplementary Figure 6

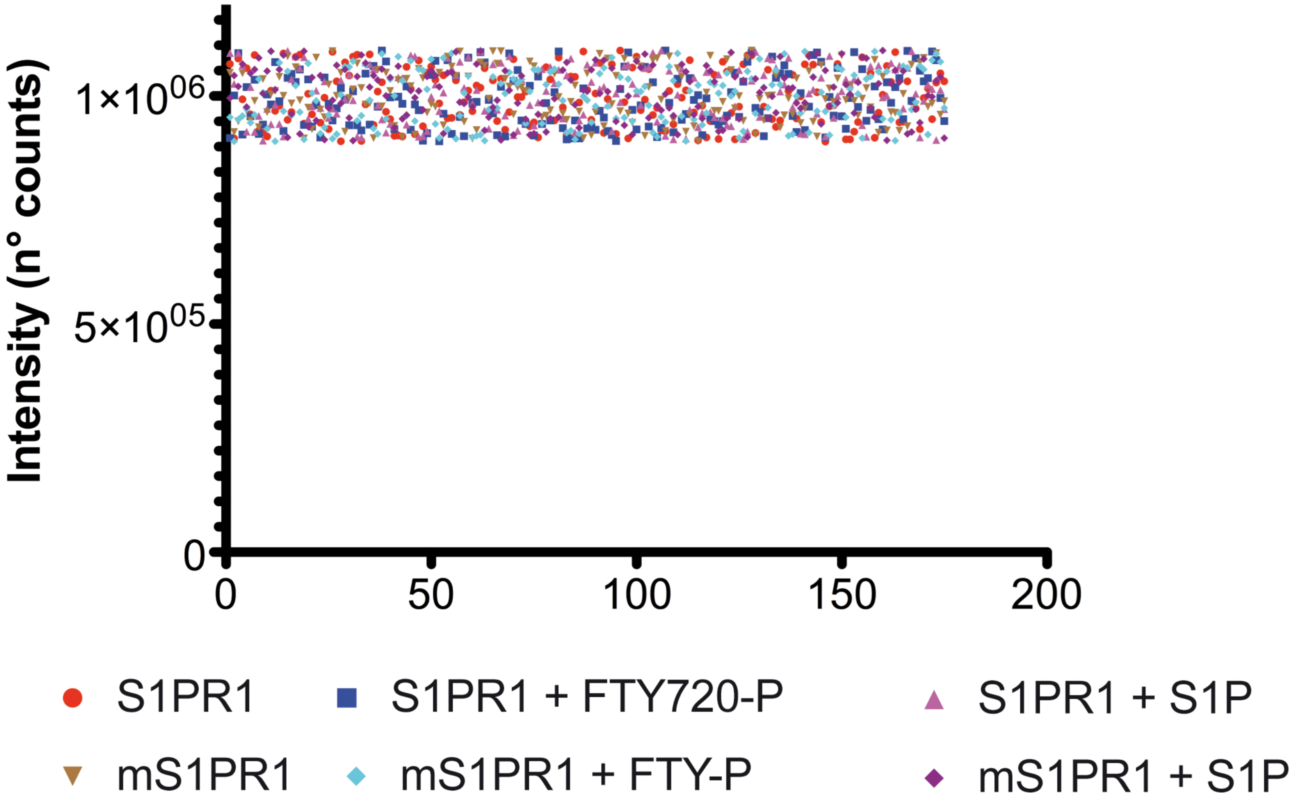

Supplementary Figure 6 | Intensity of individual DLS measurements showed in Fig. S4 E.

Supplementary Figure 7

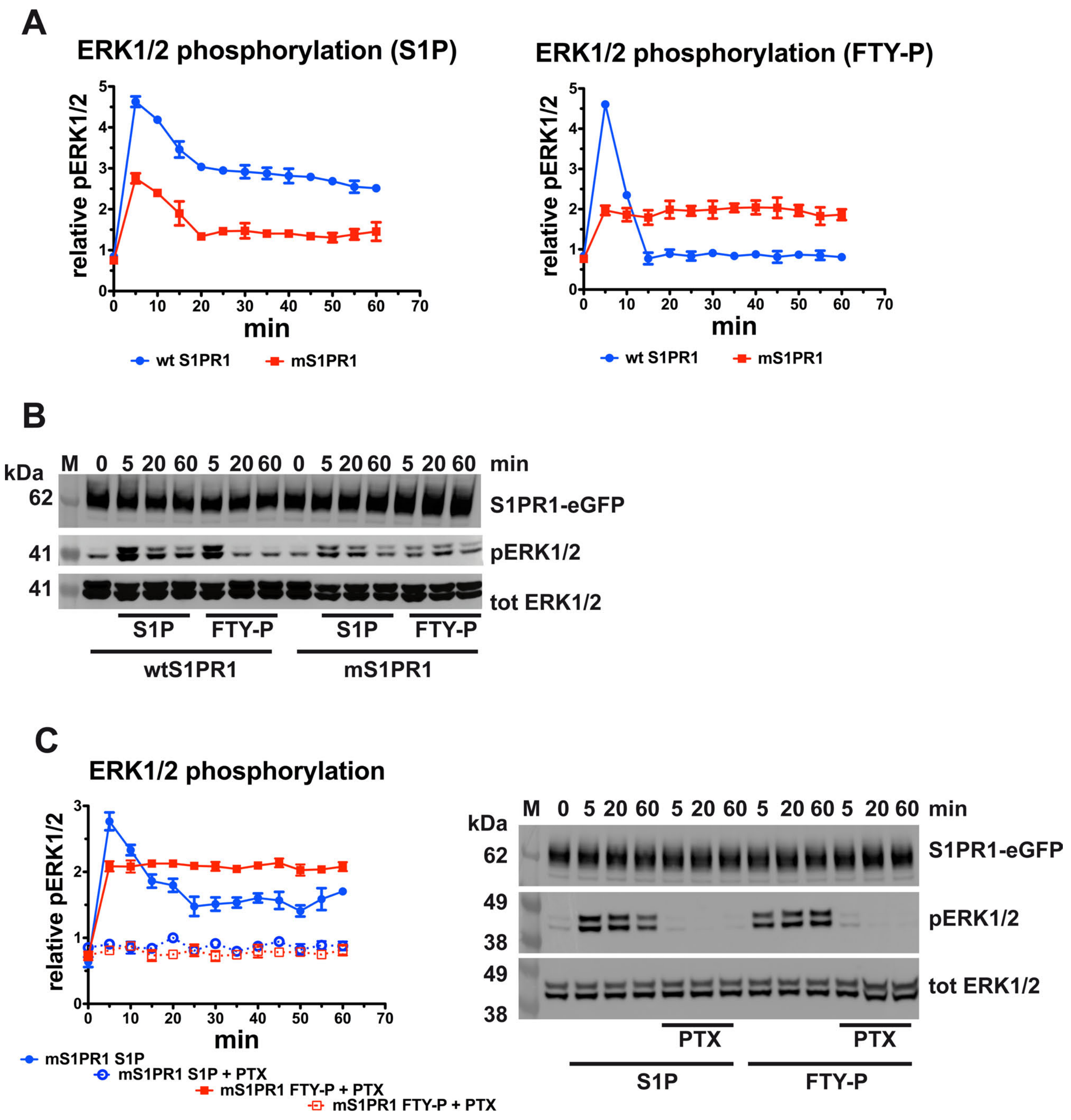

Supplementary Figure 7 | Signalling from mS1PR1-eGFP (a) S1P-starved HEK293 cells expressing either S1PR1-eGFP or mS1PR1-eGFP were stimulated with 100 nM S1P or FTY720-P, processed and analysed as in (Fig. 1 A); n=3 independent experiments. **(b)** Cells as in (a) processed for S1PR1, ERK1/2 and pERK1/2 immunoblotting. **(c)** S1P-starved HEK293 cells expressing mS1PR1-eGFP were treated with or without 300 ng/mL pertussis

toxin (PTX) for 18 hours and stimulated with 100 nM S1P or FTY720-P, processed and analysed as in (a); n=3 independent experiments.

Supplementary Figure 8

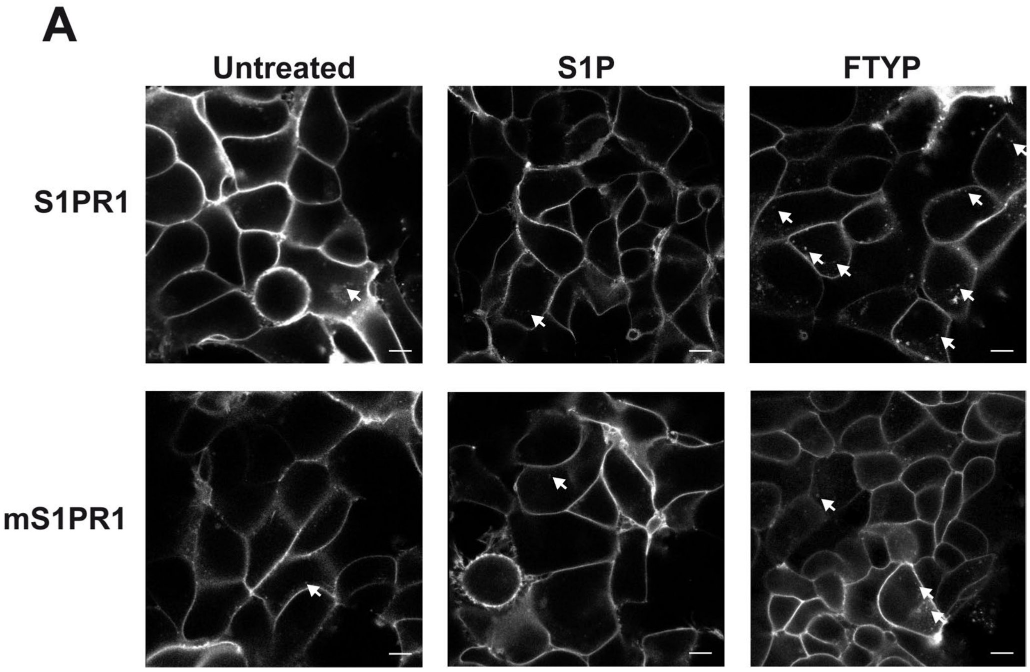

Supplementary Figure 8 | Representative mid-plane confocal images of samples analysed as shown in Fig. 4 A; arrows point to clustered (m)S1PR1-eGFP+.

Supplementary Figure 9

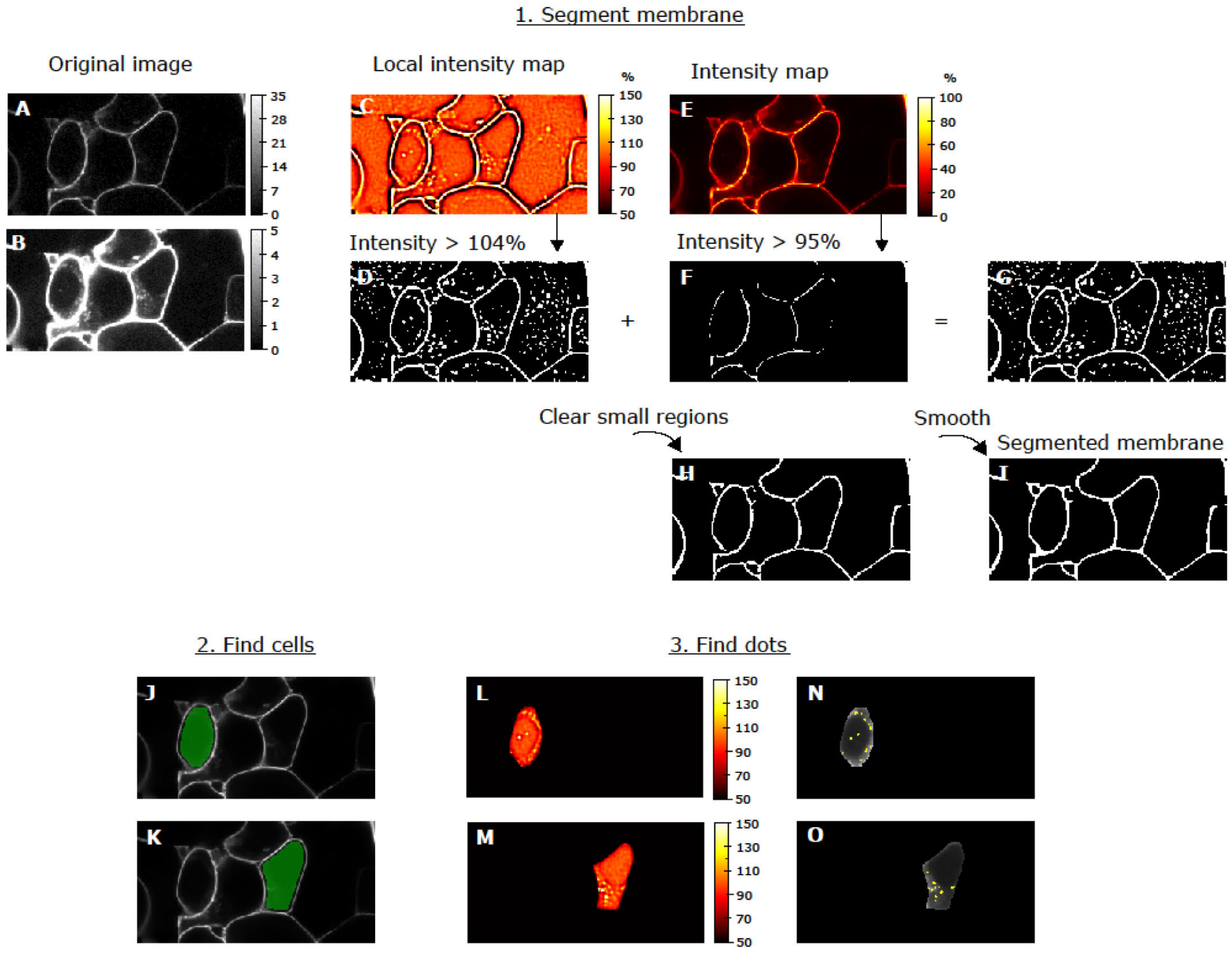

Supplementary Figure 9 | Workflow of the operations executed for the automated identification of intracellular vesicles. (**a, b**). Example of raw images used for the analysis, displayed with two different intensity ranges. Bright vesicles/dots inside the cell are counted by segmenting the cell membranes (**c-i**), followed by identifying the cellular spaces completely enclosed in membranes (**j, k**) and by and counting dots cell by cell (**l, o**). In more detail, the cellular membranes are identified by generating two separate masks that are then merged together. In the first mask (**c, d**), local maxima pixels are identified with an edge preserving filter as those higher than 104% of the local background –as estimated from a Gaussian smoothed version of the image (kernel: 7x7). The second mask contains the 5% brightest pixels of the image (**e, f**). Following merging of the two masks (**g**), removal of

objects smaller than 50 pixels (**h**) and smoothing of the resulting mask (**i**), the interior of the cell is identified as those areas completely enclosed into membranes (**j, k**). For each of the segmented cell interior, we count the number of vesicles as the number of local maxima, identified as the pixels higher than the 115% of the local background (**l-o**).

Supplementary Figure 10

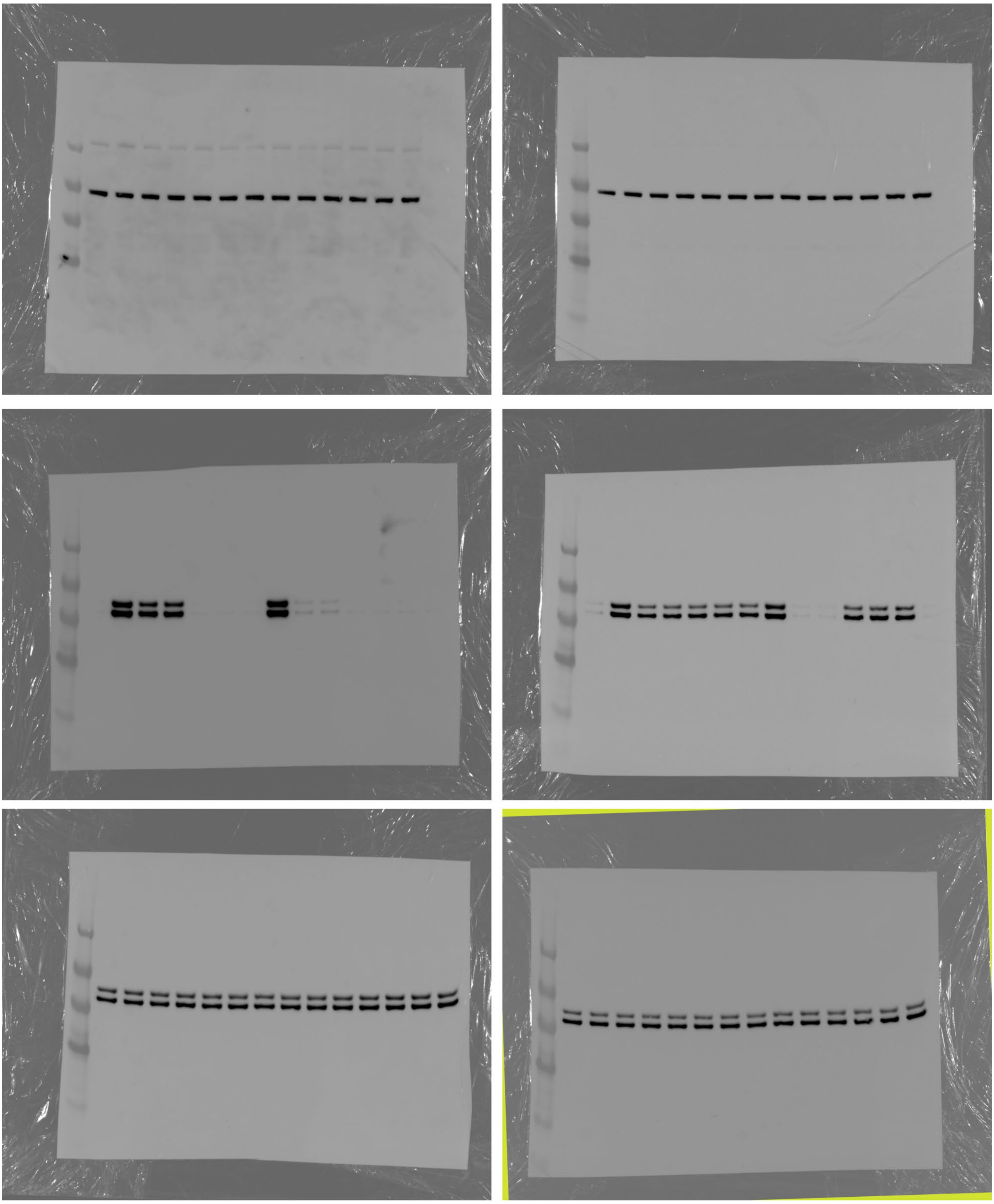

Uncropped immublots for Figure 1

Uncropped immublots for Figure 2

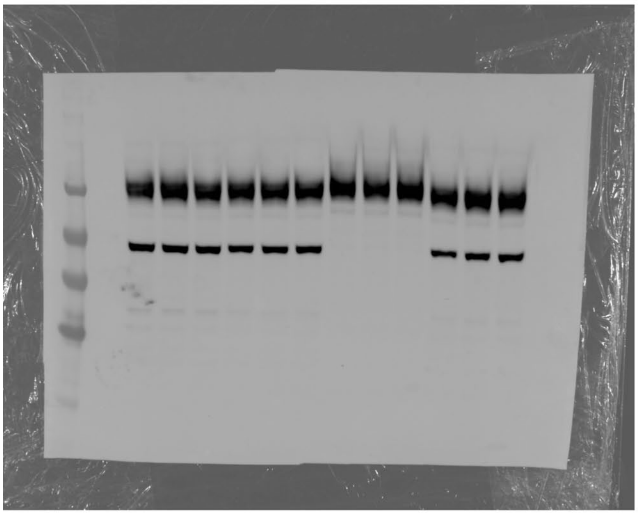

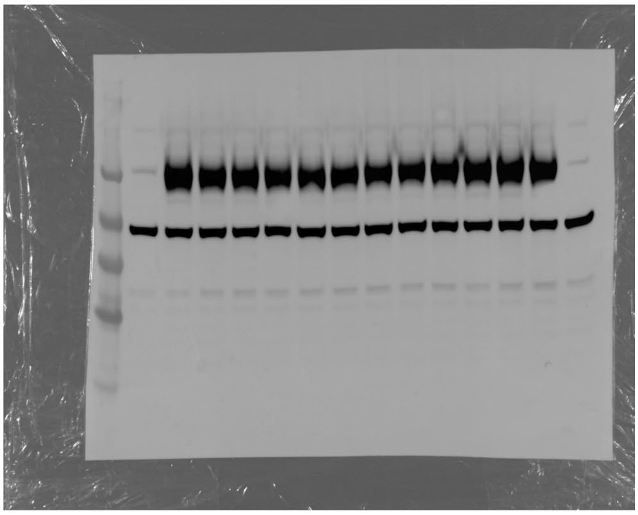

Uncropped immublots for Figure 3 D

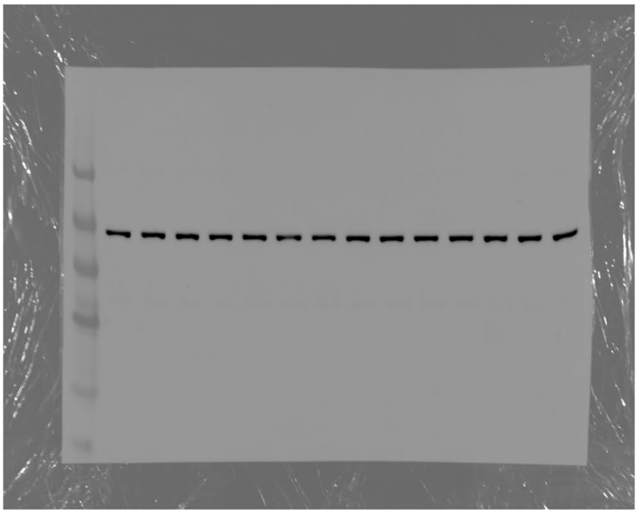

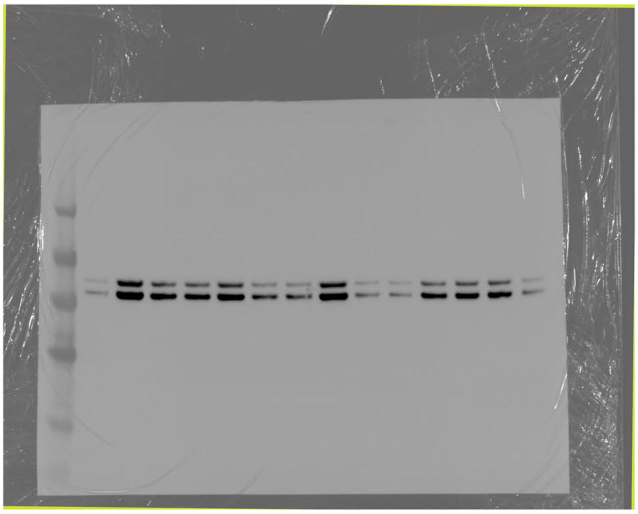

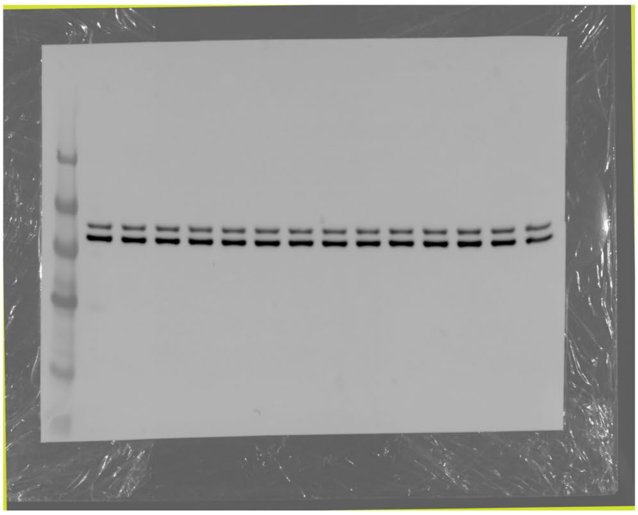

Uncropped immublots for Figures 3 E,F

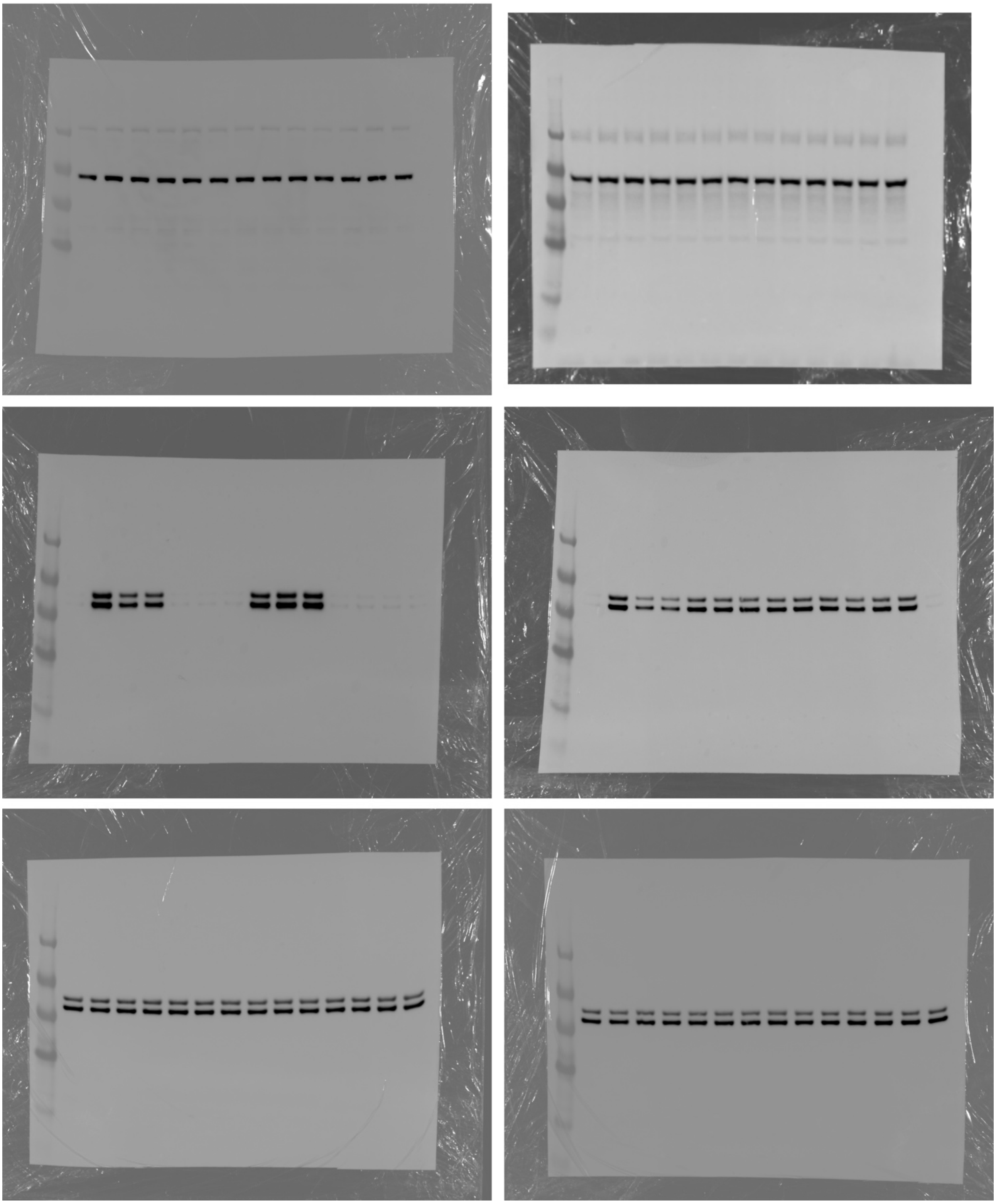

Uncropped immublots for Figure 4 C

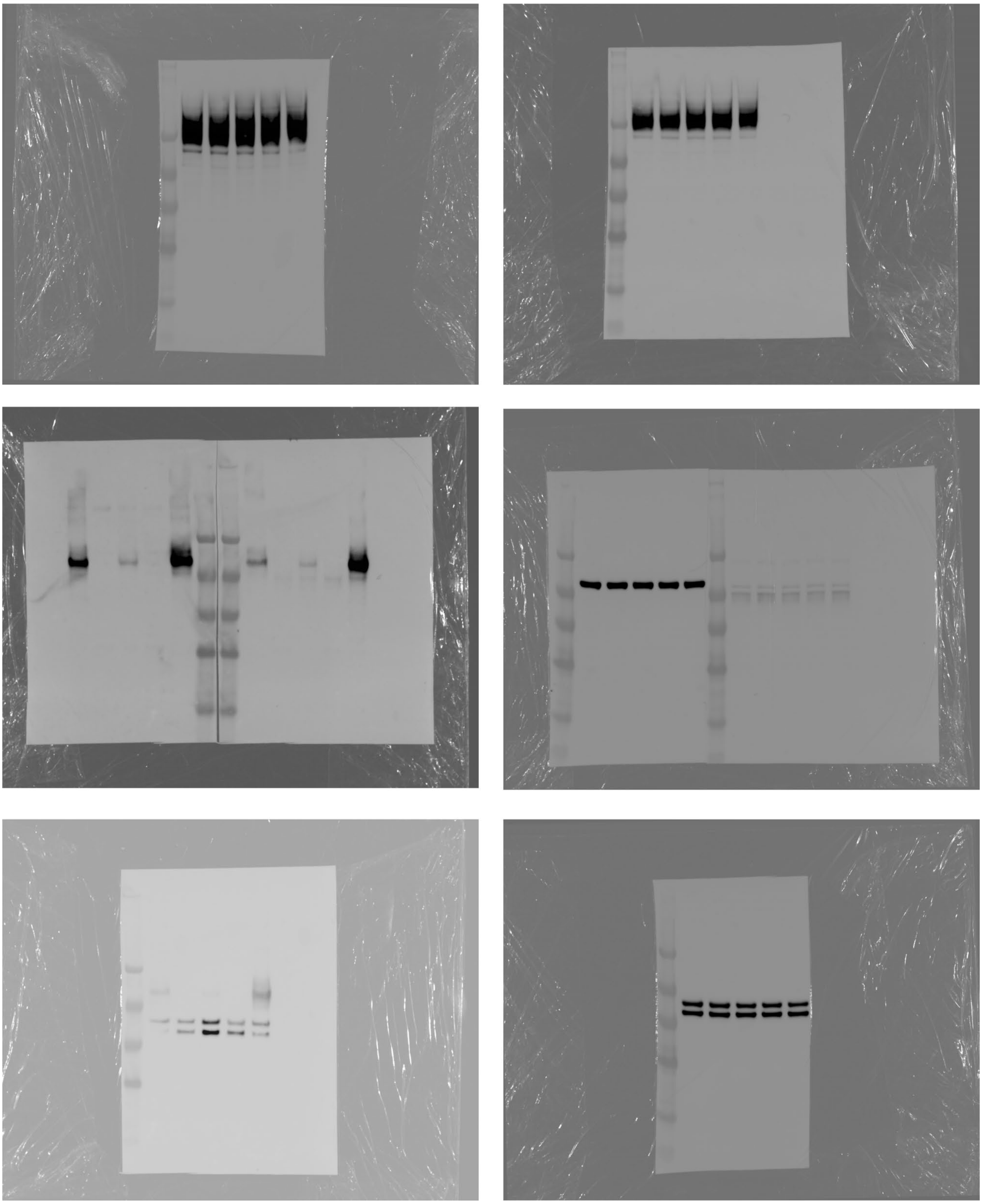

Uncropped immublots for Figure 4 E

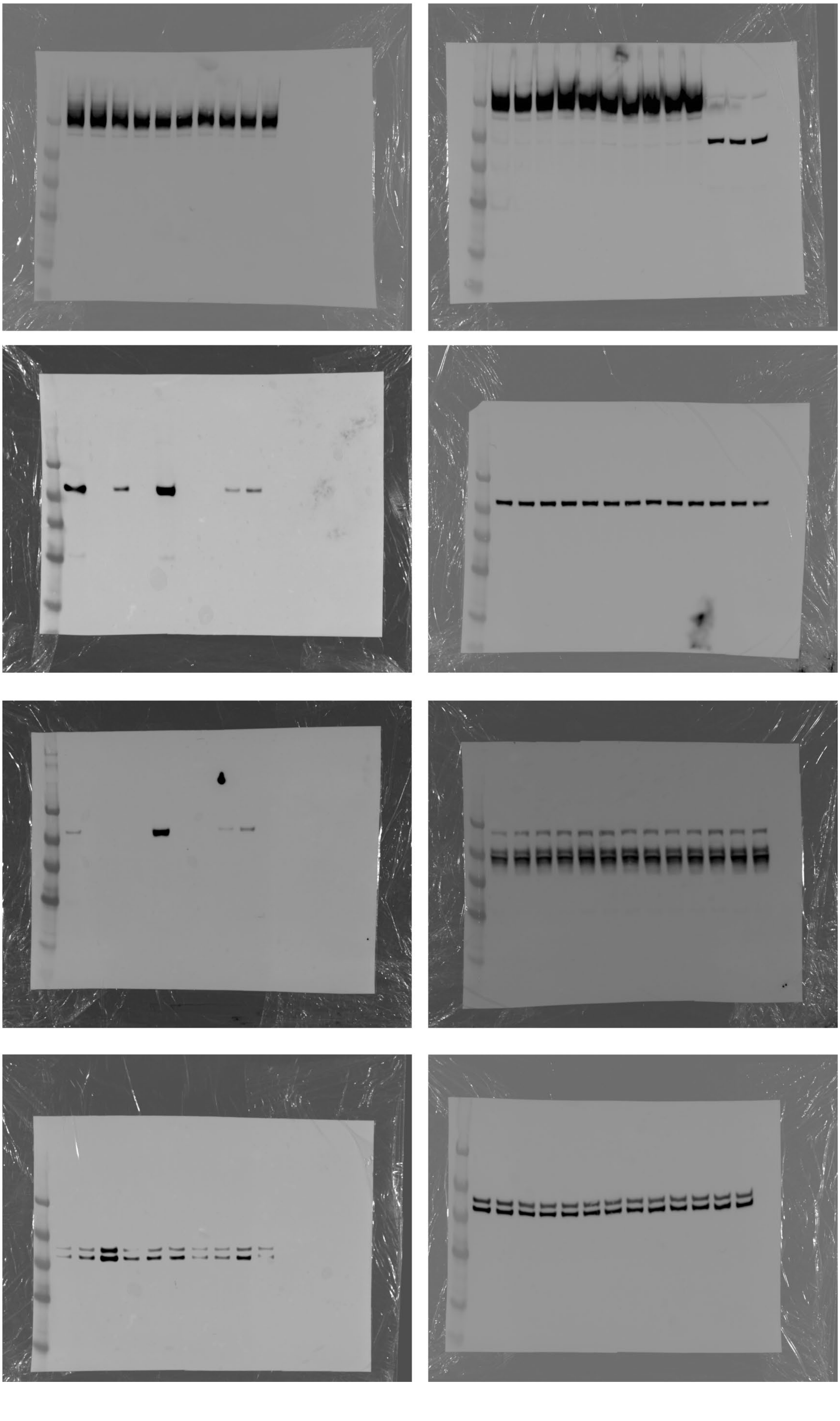

Uncropped immublots for Supplementary Figure 2 C

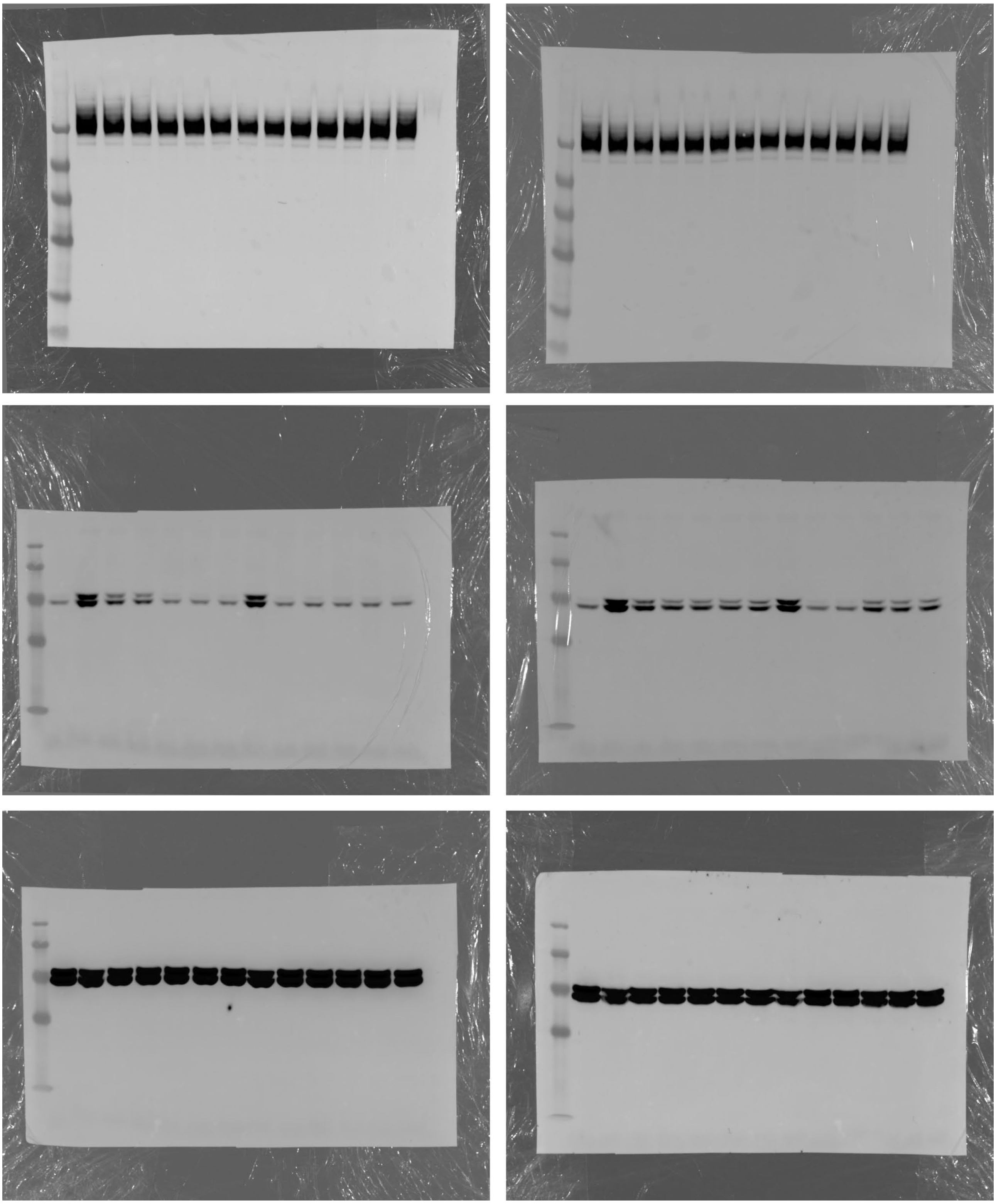

Uncropped immublots for Supplementary Figure 2 D

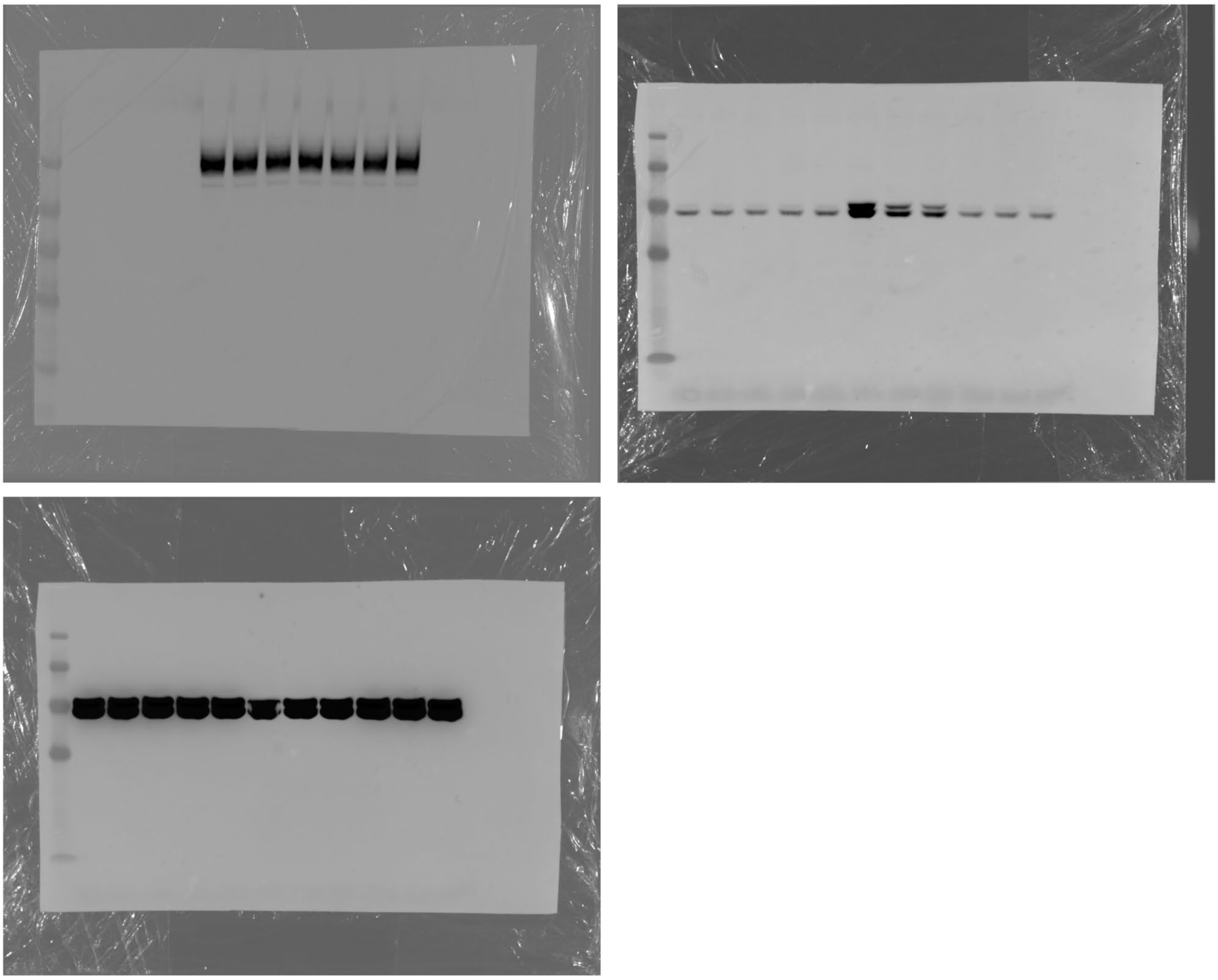

Uncropped immublots for Supplementary Figure 4

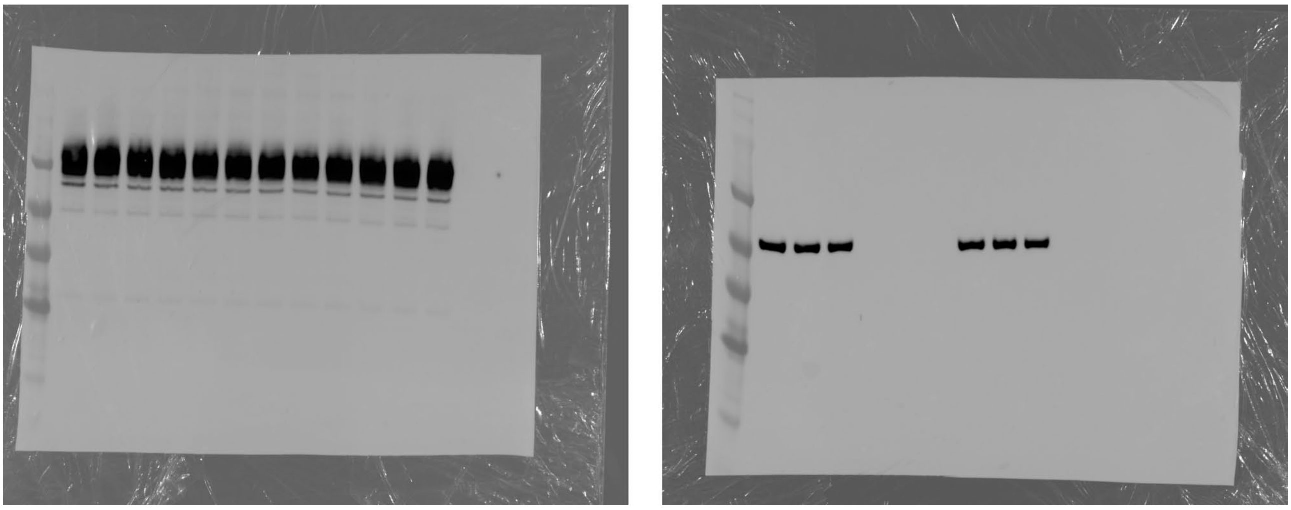

Uncropped immublots for Supplementary Figure 7

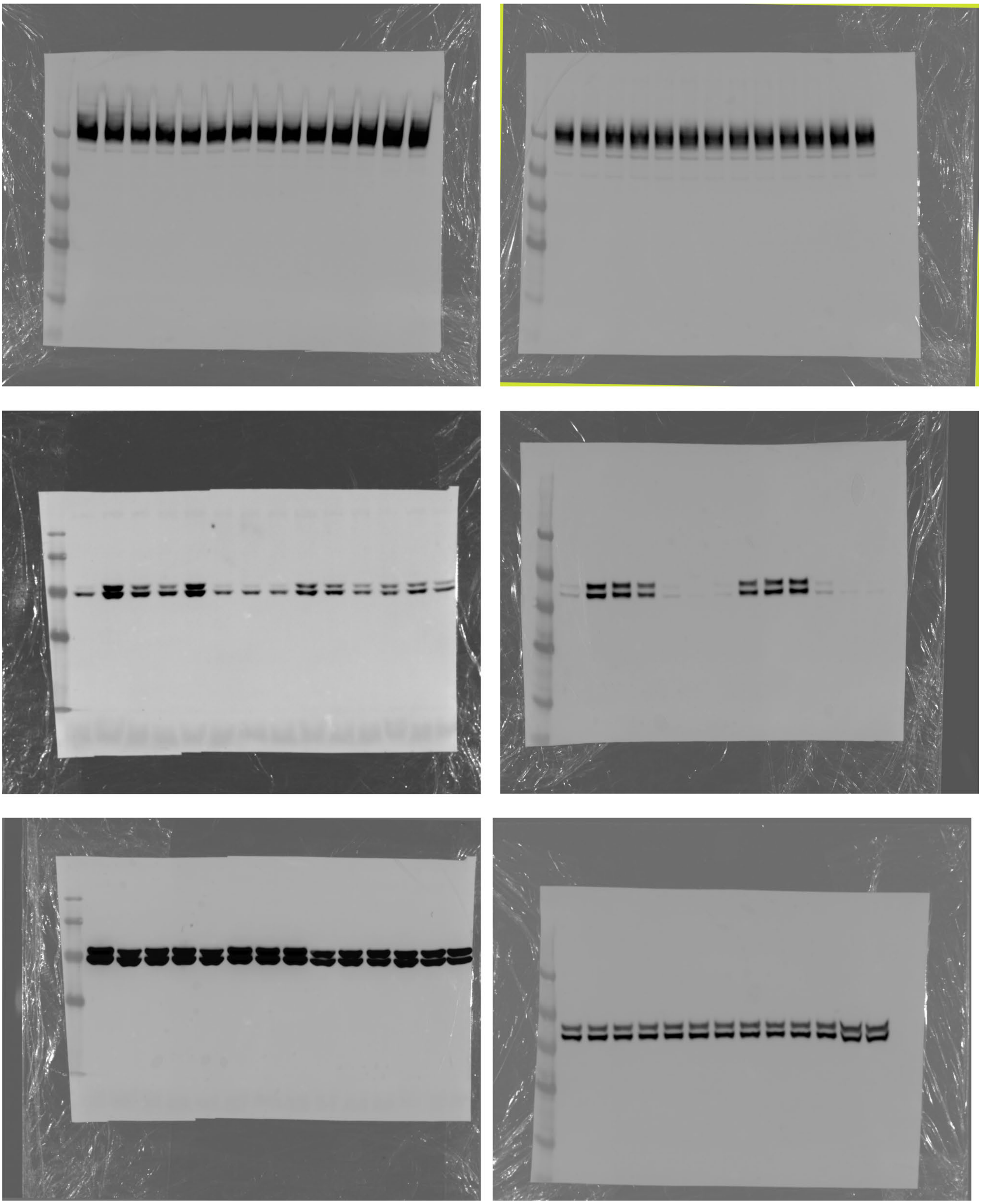

Supplement: Supplementary file 1 — Supplementary Information [file 42003_2020_752_MOESM1_ESM.pdf]
