# Supplementary material for: Combinatorial allosteric modulation of agonist response in a self-interacting G-protein coupled receptor
Source: Commun Biol. 2020 Jan 15;3:27. doi: 10.1038/s42003-020-0752-4 (PMC6962373; doi:10.1038/s42003-020-0752-4)
Supplement: Supplementary file 2 — Description of Additional Supplementary Files [file 42003_2020_752_MOESM2_ESM.docx]

**Supplementary Data 1.** MATLAB script used for the automated determination of the number of eGFP^+^ intracellular “dot-like” structures from confocal microscopy images of HEK293 cells expressing S1PR1 and its variants.
