# Supplementary material for: Combinatorial allosteric modulation of agonist response in a self-interacting G-protein coupled receptor
Source: Commun Biol. 2020 Jan 15;3:27. doi: 10.1038/s42003-020-0752-4 (PMC6962373; doi:10.1038/s42003-020-0752-4)
Supplement: Supplementary file 4 — Reporting Summary [file 42003_2020_752_MOESM4_ESM.pdf]

nature research

Corresponding author(s): Massimo Degano

Last updated by author(s): 2019/12/09

Reporting Summary

Nature Research wishes to improve the reproducibility of the work that we publish. This form provides structure for consistency and transparency in reporting. For further information on Nature Research policies, see Authors & Referees and the Editorial Policy Checklist.

Statistics

For all statistical analyses, confirm that the following items are present in the figure legend, table legend, main text, or Methods section.

n/a	Confirmed	
☐	☒	The exact sample size (n) for each experimental group/condition, given as a discrete number and unit of measurement
☐	☒	A statement on whether measurements were taken from distinct samples or whether the same sample was measured repeatedly
☐	☒	The statistical test(s) used AND whether they are one- or two-sided<br>*Only common tests should be described solely by name; describe more complex techniques in the Methods section.*
☒	☐	A description of all covariates tested
☒	☐	A description of any assumptions or corrections, such as tests of normality and adjustment for multiple comparisons
☐	☒	A full description of the statistical parameters including central tendency (e.g. means) or other basic estimates (e.g. regression coefficient) AND variation (e.g. standard deviation) or associated estimates of uncertainty (e.g. confidence intervals)
☐	☒	For null hypothesis testing, the test statistic (e.g. F, t, r) with confidence intervals, effect sizes, degrees of freedom and P value noted<br>*Give P values as exact values whenever suitable.*
☒	☐	For Bayesian analysis, information on the choice of priors and Markov chain Monte Carlo settings
☒	☐	For hierarchical and complex designs, identification of the appropriate level for tests and full reporting of outcomes
☒	☐	Estimates of effect sizes (e.g. Cohen's d, Pearson's r), indicating how they were calculated

Our web collection on statistics for biologists contains articles on many of the points above.

Software and code

Policy information about availability of computer code

Data collection	Image Lab v5.2.1, Cary Eclypse Scan v1.0.0.610, Magellan v6.4, Dynamics v6, LAS X
Data analysis	GraphPad Prism v5.0, PyMOL v2.2.0, PISA web server, FoldX web server, Huygens Professional Software v17.04, custom MatLab script

For manuscripts utilizing custom algorithms or software that are central to the research but not yet described in published literature, software must be made available to editors/reviewers. We strongly encourage code deposition in a community repository (e.g. GitHub). See the Nature Research guidelines for submitting code & software for further information.

Data

Policy information about availability of data

All manuscripts must include a data availability statement. This statement should provide the following information, where applicable:

- Accession codes, unique identifiers, or web links for publicly available datasets
- A list of figures that have associated raw data
- A description of any restrictions on data availability

NCBI reference sequence NP_001391, Uniprot accession code P21453 and PDB code 3V2Y were used in this study. Figures with associated raw data: Fig. 1,2,3,4,6,S2,S5,S6,S7. Materials and all other data are available from patrone.marco@hsr.it or degano.massimo@hsr.it upon reasonable request

Field-specific reporting

Please select the one below that is the best fit for your research. If you are not sure, read the appropriate sections before making your selection.

☒ Life sciences ☐ Behavioural & social sciences ☐ Ecological, evolutionary & environmental sciences

Life sciences study design

All studies must disclose on these points even when the disclosure is negative.

Sample size	Sample sizes were chosen consistently to the type of the experiments and regarded as sufficient based on the standard deviations and the profile coherence (ELISA tests and binding curves) or because they were an extensive characterization of the samples without any degradation of the signal intensity (DLS). Endocytic vesicle counting was totally unsupervised and the number of cells for each condition tested was solely dependent on the activity by the script to identify membrane closed areas (as "cell" was defined in the script) from the fluorescence micrographs, by this means independently sampling the cohort of cells recorded in the different fields corresponding to the data points.
Data exclusions	no data were excluded
Replication	All quantitative measures were performed as independent replicates. All attempts to replicate the data were consistent.
Randomization	Randomization was not relevant to this study as all the measures were recorded integrally by automatic equipments and no manual counting was performed by the experimenters
Blinding	Blinding was not relevant to this study as all the measures were recorded integrally by automatic equipements and no manual counting was performed by the experimenters

Reporting for specific materials, systems and methods

We require information from authors about some types of materials, experimental systems and methods used in many studies. Here, indicate whether each material, system or method listed is relevant to your study. If you are not sure if a list item applies to your research, read the appropriate section before selecting a response.

Materials & experimental systems

n/a	Involved in the study	
☐	☒	Antibodies
☐	☒	Eukaryotic cell lines
☒	☐	Palaeontology
☒	☐	Animals and other organisms
☒	☐	Human research participants
☒	☐	Clinical data

Methods

n/a	Involved in the study	
☒	☐	ChIP-seq
☒	☐	Flow cytometry
☒	☐	MRI-based neuroimaging

Antibodies

Antibodies used

Rabbit poly-clonal anti-S1PR1, Abcam, cat.n. ab137467, lot GR103634-15, dilution 1:1000
Rabbit monoclonal anti-β-arrestin 1, Cell Signaling Technology, cat. n. 12697, clone D8O3J, lot 1, dilution 1:1000
Rabbit monoclonal anti-β-arrestin 2, Cell Signaling Technology, cat. n. 3857, clone C16D9, lot 2, dilution 1:1000
Rabbit monoclonal anti-p42/p44 MAPK (ERK1/2), Cell Signaling Technology, cat. n. 4695, clone 137F5, lot 21, dilution 1:1000
Rabbit monoclonal anti-phospho-p42/p44 MAPK (ERK1/2) (Thr202/Tyr204), Cell Signaling Technology, cat. n. 4370, clone D13.14.4E, lot 17, dilution 1:2000
Rabbit poly-clonal anti-LPAR1, Abcam, cat.n. ab84788, lot GR57094-1, dilution dilution 1:1000

Validation

Rabbit poly-clonal anti-S1PR1 manufacturer's statement: immunogen affinity purified, immunoblot on: Hep G2 whole cell lysate, mouse brain tissue, purified S1PR1
Rabbit monoclonal anti-β-arrestin 1 manufacturer's statement: it recognizes endogenous levels of β-arrestin 1 by immunoblot in MDA-MB-425, Hep G2 PANC-1 cell lines; it immunoprecipitates β-arrestin 1 from LN18 cell extracts against rabbit mAb isotype control (clone DA1E); it does not cross-react with β-arrestin 2
Rabbit monoclonal anti-β-arrestin 2 manufacturer's statement: it recognizes endogenous levels of β-arrestin 2 by immunoblot in HeLa, NIH/3T3, A10, COS and Jurkat cell extracts
Rabbit monoclonal anti-p42/p44 MAPK (ERK1/2) manufacturer's statement:it detects endogenous levels of total p42/p44 MAPK (ERK1/2) by immunoblotmin HeLa, NIH/3T3 and C6 cell extracts; it does not cross-react with JNK/SAPK or p38 MAP kinase
Rabbit monoclonal anti-phospho-p42/p44 MAPK (ERK1/2) (Thr202/Tyr204) manufacturer's statement: it detects endogenous levels of total p42/p44 MAPK (ERK1/2) when dually phosphorylated at Thr202/Tyr204 of Erk1 (Thr185 and Tyr187 of ERK2), and singly phosphorylated at Thr202 in COS cell extracts in treated or not with TPA, with or without U0126 MEK 1/2 inhibitor as negative control; it does not cross-react with the corresponding phosphorylated residues of either JNK/SAPK or p38 MAP kinase
Rabbit poly-clonal anti-LPAR1 manufacturer's statement: immunogen affinity purified, immunoblot on: Jurkat, HeLa, Caco 2, A375 cell lysates

Eukaryotic cell lines

Policy information about cell lines

Cell line source(s)	HEK 293: ATCC
Authentication	Cells were not authenticated
Mycoplasma contamination	Cell lines were tested negative for mycoplasma contamination
Commonly misidentified lines (See ICLAC register)	None
